# Assessment of the efficacy of a gonadotropin releasing factor (GnRF) analog to suppress ovarian function in gilts under pre-clinical and clinical conditions

**DOI:** 10.1186/s40813-024-00383-9

**Published:** 2024-09-30

**Authors:** Sandra Genís, Vickie King, Marijke Aluwé, Alice Van den Broeke, Frédéric Descamps, Álvaro Aldaz, Niels Wuyts, Alícia Reixach, Mònica Balasch

**Affiliations:** 1 Zoetis Manufacturing & Research Spain S.L, Vall de Bianya, Spain; 2 Zoetis Inc., Parsippany, USA; 3 Institute of Agricultural, Fisheries and Food Research (ILVO), Melle, Belgium; 4 Zoetis Belgium S.A., Zaventem, Belgium

**Keywords:** Gonadotropin releasing factor (GnRF), anti-GnRF analog, Gilts, Ovarian function

## Abstract

**Background:**

The administration of a gonadotropin releasing factor (GnRF) analog to pigs has proven to induce antibodies against endogenous GnRF. In gilts (young female pigs), the subsequent blocking of GnRF activity by specific antibodies results in a temporary suppression of ovarian activity and sexual maturation. One pre-clinical and two clinical studies were conducted to assess the ability of the GnRF analog to produce immunologically suppression of the ovarian function, preventing gilts from reaching puberty before harvest, at 27 weeks of age.

**Results:**

In the three studies, a significant reduction of size and weight of reproductive organs and gilts in oestrus was demonstrated in vaccinated gilts compared with intact gilts. A significant increase in anti-GnRF antibody levels in sera was observed after the 2nd dose, which lasted until the end of the study in each of the protocols used. Progesterone levels were significantly reduced from 6 to 8 weeks after 2nd vaccination in clinical studies 2 and 1 respectively, and from 6 weeks after 2nd vaccination in the pre-clinical study. Estradiol levels were below the limit of detection for clinical study 2 and significantly reduced in vaccinated gilts at the end of the pre-clinical study and the clinical study 1.

**Conclusions:**

Vaccination of gilts with a GnRF analog with two different protocols (1st dose from 10 to 14 weeks of age, and a 2nd dose 8 or 4 weeks later) was effective in reducing the development of puberty for at least 9 weeks post 2nd dose. These results confirm the flexibility of vaccination programs for veterinarians and producers which can be adapted to pig management practices in commercial farms.

## Background

Several million entire male pigs are raised every year in Europe. Commingling entire male and female pigs in fattening pens is a common practice. In mix gender pens, entire male pigs display mounting behaviors as they mature sexually, which may result in injuries, broken legs, skin lesions, and reduction of carcass quality [1–3]. Moreover, there is a risk for unwanted pregnancies. Physical castration of young male piglets is also the most common practice to deal with the problem of boar taint in entire male pigs, which is caused by androsterone and skatole. As a side-effect, it also handles the challenges related to entire male pig behavior and unwanted pregnancies. It is commonly performed in male pigs, but in some regions like the south of Spain where Iberian pigs are raised in outdoor extensive conditions [4], it has been performed in young female pigs. In many cases, physical castration is still carried out without pain treatment and/or anesthesia [5], and it has become an animal welfare issue increasing general public concerns regarding the pain associated with traditional physical castration. Therefore, alternatives have been developed, like immunocastration, an immunization against GnRF.

The gonadotropin releasing factor (GnRF), also known as gonadotropin releasing hormone (GnRH), is a 10 amino acid-long peptide produced in the hypothalamus that stimulates the synthesis and release of the follicular-stimulating hormone (FSH) and the luteinizing hormone (LH) from the anterior pituitary gland. In males as well as females, FSH and LH are the key gonadotrophic hormones that regulate the testicular and ovarian development and function respectively, with these endocrine mechanisms being highly conserved across mammals and most vertebrates [6, 7]. Blocking the endogenous GnRF with anti-GnRF antibodies causes hypothalamic hypogonadism and inhibits sexual maturation and function in males and females across mammalian species [8–11].

There are some anti-GnRF commercial vaccines [12] available that are authorized for use in males as an alternative to physical castration for the reduction of boar taint, and to reduce aggressive and sexual behaviors. However, until recently, there were no anti-GnRF vaccines commercially available authorized for an equivalent use in females in Europe. The only exception was a product solely for use in Iberian female pigs [9].

To assess the ability of anti-GnRF vaccine to prevent sexual behavior (standing oestrus) in gilts destined for slaughter, three different studies were conducted: a preclinical efficacy study and two clinical studies to assess the efficacy of an anti-GnRF analog in suppressing ovarian function and puberty in gilts slaughtered at 26 or 27 weeks of age.

## Methods

### Ethics statement

The pre-clinical study was conducted in the facilities of Zoetis Manufacturing & Research Spain S.L. (Olot, Spain), whereas the two clinical studies were conducted at a commercial farm representative of the pig industry in Belgium (ILVO, Melle, Belgium). All experimental procedures were approved by the Zoetis Animal Welfare Committee and the competent authorities.

### Animals and experimental design

All studies were performed double blinded. The pre-clinical study was performed earlier in time with a reduced number of animals and in a control environment, where safety and efficacy parameters were evaluated. Clinical studies were performed later, in farm conditions, with a larger number of animals evaluating only efficacy parameters. For the purpose of this article, only the efficacy parameters were discussed, in order to compare the three studies.

**Fig. 1:**
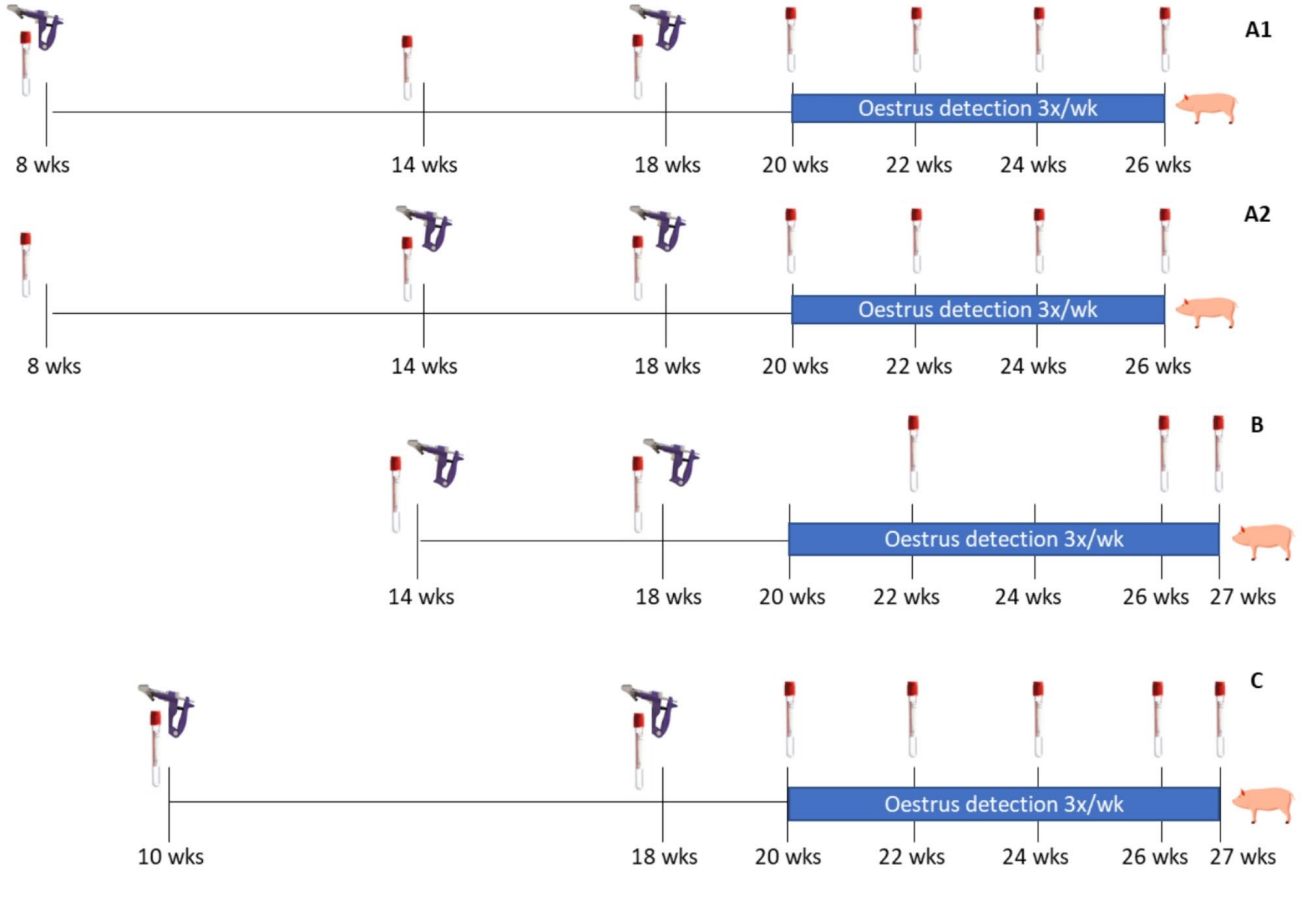
Experimental design of the studies including timing of vaccination (safety gun), blood sampling (blood tube), oestrus detection, and slaughter (pig icon) and: (**A**) pre-clinical study (*n* = 57) with early (A1) and late priming (A2), (**B**) the 1st clinical study with late priming (*n* = 80), and (**C**) 2nd clinical study with early priming (*n* = 40)

*Pre-clinical study* (Fig. 1A1, 1A2): fifty-seven clinically healthy, cross-bred gilts were enrolled at 56 days of age (8 weeks of age) and randomly allocated to one of three treatments (control, early priming [Fig. 1A] or late priming [Fig. 1B]). All animals were vaccinated at 8, 14 and 18 weeks of age either with saline or with the anti-GnRF analog (Improvac^®^, Zoetis Belgium SA, Belgium; 306 μg/dose). Control animals always received saline. Early priming animals were vaccinated with anti-GnRF analog at 8 and 18 weeks of age and with saline at 14 weeks of age; and late priming gilts with saline at 8 weeks of age and with anti-GnRF analog at 14 and 18 weeks of age. All treatment groups were commingled throughout the study. Vaccination was performed with 2 mL of either saline or anti-GnRF analog (Improvac^®^, Zoetis Belgium SA, Belgium) subcutaneously with a 16 G ¾ needle and a Simcro Sekurus safety device (Simcro, UK). They were administered in the right upper side of the neck, left side of the neck, and right lower side of the neck for the 1st, 2nd and 3rd administration respectively.

*Clinical studies 1 and 2* (Fig. 1B C): clinical study 1 enrolled eighty fattening cross-bred gilts, and clinical study 2 enrolled forty fattening cross-bred gilts. In both studies, gilts were randomly allocated into two treatment groups (control or anti-GnRF analog). Two doses of anti-GnRF analog or saline were administered at two different inter-dose intervals (IDI): 14 and 18 weeks of age (clinical study 1: four-week IDI), or 10 and 18 weeks of age (clinical study 2: eight-week IDI). Both treatment groups were commingled throughout the studies. Vaccination was performed with 2 mL of either saline or anti-GnRF analog subcutaneously with a 16 G ¾ needle and a Simcro Sekurus safety device (Simcro, UK). They were administered in the right side of the neck, and left side of the neck for the 1st and 2nd administration respectively.

All animals were fed with an age-appropriate feed, and both feed and water were provided *ad libitum*.

### Measurements and sample collection

*Oestrus detection*: gilts were observed three times per week for signals of oestrus from two weeks after the last administration until the end of each study (26 weeks of age for the pre-clinical study, and 27 weeks of age for the clinical studies). Animals from each pen were moved to a central area, where 1 out of 3 adult boars was present. Boars were alternated per oestrus check. After a 5-minute accustoming period, signs of standing oestrus were checked with every gilt. Oestrus detection was assessed by rubbing the flank and pressing the back of the gilt in the presence of the boar. Gilts were deemed to be in oestrus when they remained in standing response, immobile with arched back, and cocked ears.

*Serum collection*: Blood samples were collected before each vaccination and then approximately every two weeks post 2nd vaccination until the end of the study for the pre-clinical and clinical study 2. For clinical study 1, blood samples were taken before each vaccination and then every 28 days until the end of the study (included). Blood samples were kept for at least two hours at room temperature and then centrifuged for ten minutes at 2500 g ± 100 g at room temperature. The serum was stored at -80 ± 10ºC until further analysis.

*Reproductive tract examination*: at the end of each study, at slaughter, the uteri and ovaries were removed and weighed, and the length of the uterine horns was recorded. Each ovary was scored in relation with the size of the developing follicles and corpus luteum. The scoring system used: 0 (no follicles); 1 (immature follicles, 3–4 mm); 2 (mature follicles, 5–11 mm); and 3 (mature follicles and luteal tissue).

### Laboratory methods

*Anti-GnRF antibodies in serum*: measured with a noncompetitive ELISA test [9], which uses GnRF peptide as the capture phase. Dilutions of pig sera were added to plates coated with GnRF peptide. After several washes, the plates were incubated with horseradish peroxidase-labeled anti-swine immunoglobulin. Finally, 3,3’,5,5’-tetramethylbenzidine substrate was added. After a short incubation time, the enzyme reaction was stopped, and the color generated was measured spectrophotometrically. The amount of color produced was directly proportional to the level of antibodies present in the sample. The limit of quantification (18.56 U/mL) had been previously determined in a validation study. The analysis was performed in the facilities of Zoetis Inc. (Kalamazoo, USA).

*Progesterone and estradiol levels in serum*: analyzed by High Performance Liquid Chromatography (HPLC) – Mass Spectrometry (MS/MS) method. Progesterone-D_9_ and Estradiol-D_3_ were used as internal standards and swine serum as the matrix. The analytical range was from 10.0 to 10,000 pg/mL for both. The analyses were performed in the facilities of Zoetis Inc (Kalamazoo, USA).

### Statistical analysis

Data summaries and analyses were performed with a centralized data management system (SAS/STAT User’s Guide V. 9.4, SAS Institute, Cary, NC, USA). The individual animal was the experimental unit for all the statistical analyses.

The weight of the uterus, the sum of the weight of both ovaries and mean uterine horn length were analysed using a general linear mixed model. The fixed effect in the model was the treatment. The random effects were batch (for the clinical studies), block within batch (for the clinical studies) and residual. Treatment least squares mean, standard errors, 95% confidence interval, minimums and maximums were calculated. Contrasts were used to compare treatments within each study. Frequency distributions of follicle sizes (scores) were calculated for each treatment group within each study.

The presence or absence of at least one standing oestrus post-last vaccination (2nd for the clinical studies and last administration for the pre-clinical study) was analysed using the Cochran-Armitage test adjusting for block. Frequency distributions of the variable were calculated for each treatment group. Treatments within study were compared using a contrast.

At each time point, the proportions of pigs with anti-GnRF titers below the level of quantification in each treatment group were calculated. The data was logarithm transformed prior to analysis and then analyzed using a general linear mixed model for repeated measures, with treatment, time point and their interaction as fixed effects, and random effects of batch (for the clinical studies), block within batch (for clinical studies), animal within batch (for the clinical studies), block and treatment, and residual. Treatment comparisons within study were made at each time point using contrasts. For animals with GnRF antibody titers below the level of quantification, these were defined as half the level of quantification and included in the analyses. The treatment least squares means, standard error and 95% confidence intervals were back-transformed for each time point. Treatment minimums and maximums were also calculated for each time point.

The number and percentage of pigs with levels below the limit of quantification for progesterone and estradiol were calculated for each treatment and each time point. The progesterone and estradiol levels were analysed the same way as the anti-GnRF values except that they were not transformed.

## Results

### Reproductive tract

In all studies, either with the early priming or the late priming program, gilts vaccinated with anti-GnRF analog had significantly reduced uterus weight, horn length and ovarian weight (*P* < 0.05) compared with their respective control animals (Table 1).

In the pre-clinical study, most control gilts had mature follicles with (score 3, 78.0%) or without luteal tissue (score 2, 22.0%) whereas in the vaccinated groups most of the ovaries had immature (score 1, 41.0% early priming, 20.0% late priming) or no follicles (score 0, 43.0% early priming, 80.0% late priming) at all. In the clinical studies, 71.4% and 64.2% of control gilts respectively had mature follicles with or without luteal tissue whereas 90.5% and 96.2% of the vaccinated animals had no follicles.

**Table 1:** Summary or reproductive tract variables by study and treatment group

Study		Pre-clinical (*n* = 57)			Clinical study 1 (*n* = 80)		Clinical study 2 (*n* = 40)	
Treatment		Control	Early priming	Late priming	Control	Late priming	Control	Early priming
Uterus weight (g)		698 ± 55	163 ± 54*	61 ± 55*	262 ± 48	71 ± 48^¥^	270 ± 37	55 ± 30ψ
Horn length (mm)		745 ± 82	329 ± 45*	258 ± 55	911 ± 91	487 ± 109^¥^	765 ± 71	377 ± 49ψ
Ovarian weight (g)		14,6 ± 1,03	4,6 ± 0,99*	2,3 ± 0,36*	10 ± 1,3	3,4 ± 1,3^¥^	11,0 ± 0,9	3,2 ± 1,2ψ
Follicle size score (%)	0	2	43	80	0	90.48	0	96.23
	1	0	41	20	28.57	0	35.82	0
	2	22	3	0	14.29	0	19.4	0
	3	78	14	0	57.14	9.52	44.78	3.77

SE: Standard error; *Significantly different (P < 0.001) from control animals in the pre-clinical study; ^¥^ Significantly different (P = 0.014 for uterus weight; P = 0.013 for horn length; and P = 0.023 for ovarian weight) from control animals in the 1st clinical study

ψ Significantly different (P < 0.001 for all parameters) from control animals in the 2nd clinical study

Follicle size score: 0 = no follicles; 1 = immature; 2 = mature; 3 = mature and luteal tissue

### Standing oestrus

Significant differences were observed between the vaccinated groups and their corresponding control group in standing oestrus. In the pre-clinical study 15.8% and 0% of the early and late priming animals respectively showed oestrus compared with 83.3% of gilts of the control group (*P* < 0.0001). In the 1st clinical study 11.1% of late priming gilts showed oestrus compared to 40.0% in control gilts (*P* = 0.0349). Finally, none of the early primed gilts showed oestrus whereas 22.5% of the control gilts did in the 2nd clinical study (*P* = 0.0029).

**Fig. 2:**
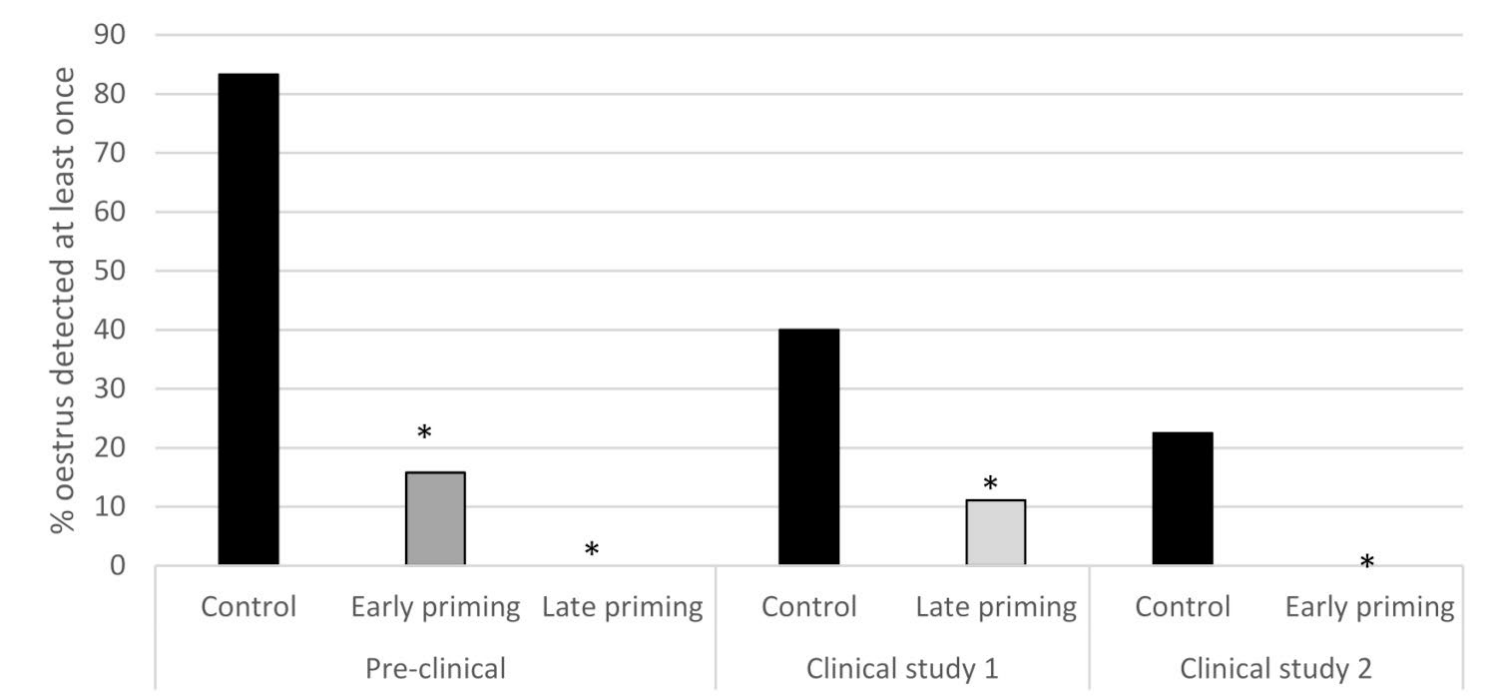
Percentage of gilts showing oestrus at least once per study and treatment group. Points with an asterisk differ (*P* < 0.05) from the control group

### Anti-GnRF antibodies

**Fig. 3:**
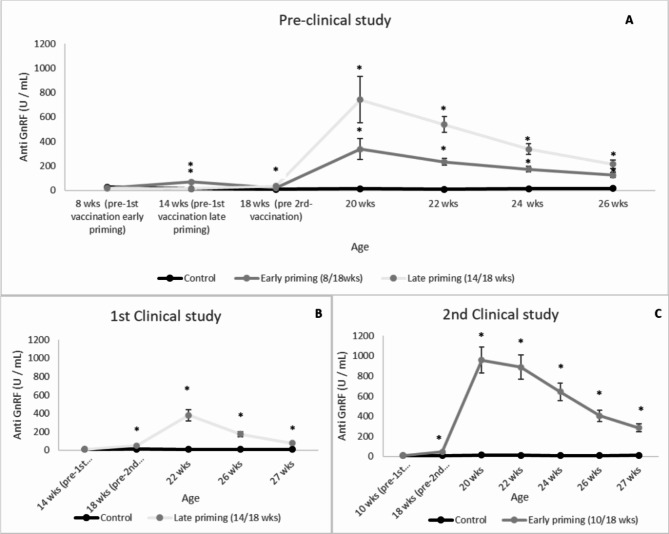
Anti-GnRF antibody results by treatment, study day and study (U/mL). (**A**) represents the results from the pre-clinical study, (**B**) from the 1st clinical study, and (**C**) from the 2nd clinical study. Points represent means ± SE. Points with an asterisk differ (*P* < 0.05) from the control group

No significant differences in anti-GnRF antibodies in serum were observed between treatments before 1st vaccination in any of the studies. Significant differences were observed from the 1st sampling after the 1st anti-GnRF analog vaccination (14 weeks of age for early priming treatment in the pre-clinical study, and from 18 weeks of age for the late priming treatment form the pre-clinical study and both clinical studies), until the end of the study. The anti-GnRF antibody results for the pre-clinical study are represented in Fig. 3A (*P* < 0.001 compared with control group); clinical study 1 in Fig. 3B (*P* < 0.001); and clinical study 2 in Fig. 3C (*P* < 0.001).

### Progesterone levels

**Fig. 4:**
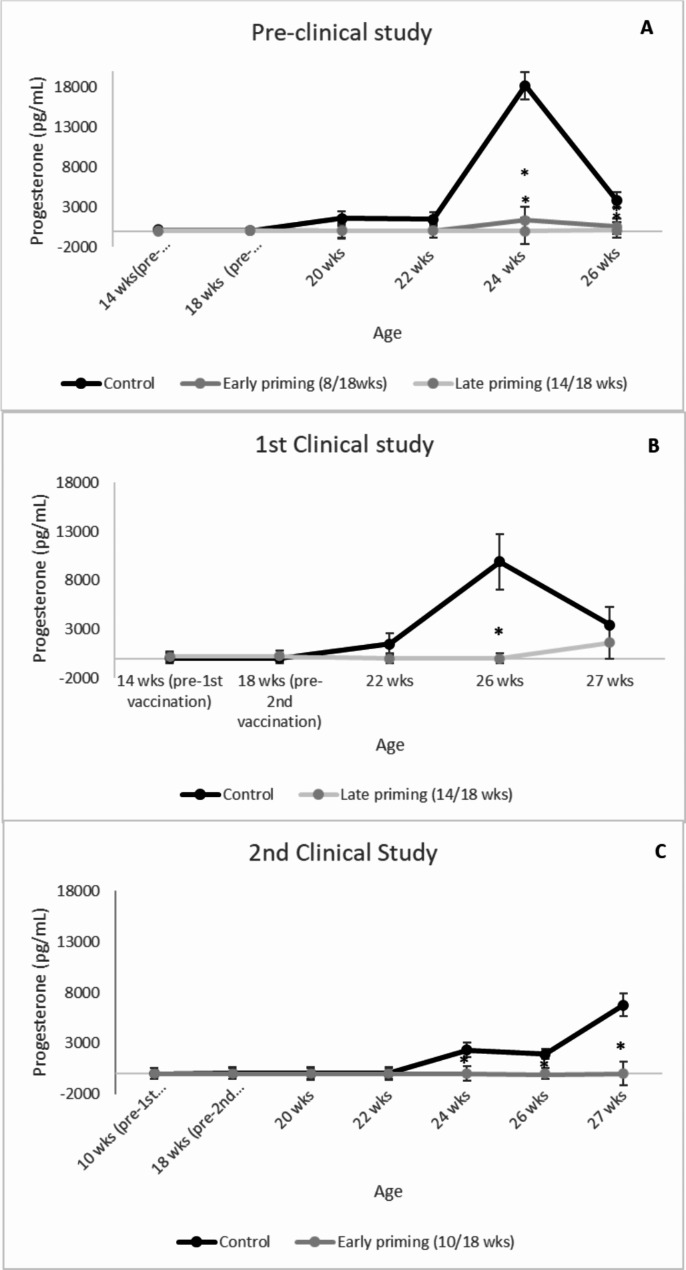
Progesterone concentrations in serum evolution by treatment, study day, and study (pg/mL). (**A**) represents the results from the pre-clinical study, (**B**) from the 1st clinical study, and (**C**) from the 2nd clinical study. Points represent means ± SE. Points with an asterisk differ (*P* < 0.05) from the control group

In all three studies, there were no significant differences in serum progesterone concentrations between treatment groups from 14 until 22 weeks of age. For the pre-clinical study, early and late primed gilts had significantly lower progesterone concentrations at 24 and 26 weeks of age (*P* = 0.017, Fig. 4A). In the 1st clinical study, progesterone levels were significantly lower at 26 wks of age (*P* < 0.001, Fig. 4B). In the 2nd clinical study, progesterone levels significantly lower from 24 weeks of age until the end of the study (27 weeks of age) (*P* < 0.05, Fig. 4C).

### Estradiol

**Fig. 5:**
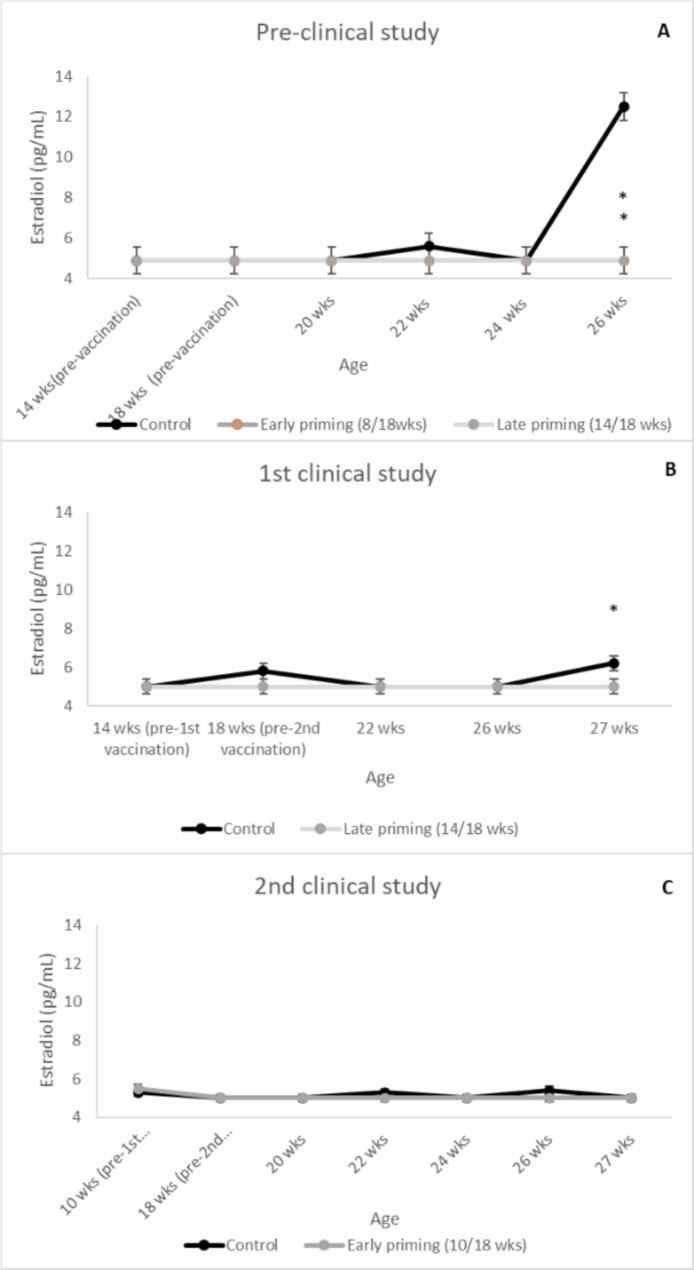
Estradiol concentrations evolution in serum by treatment, study day, and study. (**A**) represents the results from the pre-clinical study, (**B**) from the 1st clinical study, and (**C**) from the 2nd clinical study. Points represent means ± SE. Points with an asterisk differ (*P* < 0.05) from the control group

In all three studies, there were no significant differences in serum estradiol concentrations between treatment groups from 14 until 24 weeks of age. For the pre-clinical study, early and late primed gilts had significantly lower estradiol concentrations at 26 weeks of age (*P* < 0.001, Fig. 5A). In the 1st clinical study, estradiol levels were significantly lower at 27 wks of age (*P* = 0.028, Fig. 5B). In the 2nd clinical study, no significant differences on estradiol levels were observed between treatments (*P* ≥ 0.137, Fig. 4C).

## Discussion

GnRF analogs are used to induce antibodies against naturally produced GnRF with the intention to suppress the gonadal activity in fattening pigs. The two doses of the anti-GnRF analog induced a massive production of anti-GnRF antibodies in treated animals, confirmed in the three trials reported here, in contrast to the control animals. A significant increase of anti-GnRF antibody titers was observed after the 2nd dose in all anti-GnRF analog-treated gilts, which lasted until the end of each trial. Data suggests a direct correlation between the increase of anti-GnRF antibody levels in serum and the reduction of oestrus, as the inhibition of the GnRF signaling, i.e. antibodies blocking the endogenous GnRF, causing hypothalamic hypogonadism and inhibiting sexual function and maturation in both males as well as females, across mammalian species [6, 7] as well as capons [13].

Blocking GnRF activity in gilts results in a temporary suppression of the ovarian activity and sexual maturation, preventing unwanted pregnancies in situations where females are being raised commingled with entire males or due to unwanted contact with wild boars in outdoor production farms. Reproductive tract organs were indeed significantly smaller/lighter in gilts immunized with the anti-GnRF analog in the 3 studies compared to the control gilts, following both an early and late priming protocol, with a reduction of on average 56% / 65%, 47%, and 51% for the uterine horns, 77% / 91%, 73%, and 80% for the uterus weights, and 68% / 84%, 66%, and 71% for the ovarian weights (early priming/late priming pre-clinical study, 1st clinical study, 2nd clinical study, respectively). The uterine horns of mature sows typically measure between 1500 and 2000 mm [14], while one study in gilts reported a mean of 533.4 ± 176.5 mm [15]. These data are of the same order as those of the control gilts in each of the 3 studies (745 ± 65 mm pre-clinical, 911 ± 91 mm clinical study 1, and 765 ± 71 mm clinical study 2). Uterus weights of the control gilts were more deviating from literature. Steel et al. [15] reported a mean of 102.0 ± 7.9 g in uterus weight in gilts while control gilts of the current studies had uterus weights of 698 ± 55 g, 262 ± 48 g, and 270 ± 37 g for the pre-clinical, clinical 1, and clinical 2 studies respectively. The weight differences found by Steel et al. [15] and our studies may be explained by a difference in age: in Steel’s study the gilts were harvested at 160 days of age (22–23 weeks of age) whereas in our studies gilts were sampled at 26 or 27 weeks of age. Furthermore, different genetics were involved that may have also influenced the uterus weight (Large White x Landrace for Steel’s study, Large White x Landrace x Duroc for the pre-clinical study, and Pietran x hybrid sow for the clinical studies).

The aim of the vaccination with the anti-GnRF analog was to inhibit puberty onset, which is usually defined as the time of the 1st oestrus and ovulation with a continuation of a regular oestrus cycles [16, 17]. However, under clinical/pre-clinical conditions, the age at 1st observed oestrus is used to define puberty in gilts. Published articles report a range between 23 and 30 weeks of age for appearance of the fist sanding oestrus [16, 18, 19]. Control gilts started to show oestrus from 19, 21, and 22 weeks of age, with an average of 23 weeks of age for the pre-clinical study, and 25 weeks of age for the clinical studies; with a prevalence of 83.3%, 40.0% and 22.5% respectively. Immunizing with the anti-GnRF analog clearly reduced the onset of puberty in both early and late priming groups: none of the late priming animals showed standing oestrus in the pre-clinical and only 11.1% on the 1st clinical study, whereas 15.8% of early priming gilts showed oestrus at the pre-clinical study, and none in the 2nd clinical study.

These results demonstrate that the administration of an anti-GnRF analog was successful, with different degrees of efficacy, to reduce the incidence of standing oestrus in treated gilts. Based on these results, both early vaccination (10 and 18 weeks of age) and late vaccination (14 and 18 weeks of age) are able to significantly reduce the incidence of standing oestrus. These results support the flexibility with regard to the vaccination schedule which best suits management practices in commercial farms.

Results for progesterone confirm the results found for standing behaviour. Mean progesterone levels were significantly lower in the anti-GnRF analog group at the end of the study period compared to the control gilts. For the latter, levels were higher as the level of progesterone increases for approximately 10 days of the oestrus cycle which lasts 18–24 days in total [17]. The reduction of progesterone level in treated gilts confirmed the mode of action of the anti-GnRF analog as it down-regulates GnRF production and therefore, inhibits the synthesis and release of follicular-stimulating hormone (FSH) and luteinizing hormone (LH). If those hormones are not secreted for the preparation of oestrus, the corpus luteum is not formed, and consequently there is no progesterone secretion [20, 21].

Also, estradiol levels significantly differed at the end of the pre-clinical and 1st clinical study. Control animals had a significant increase of estradiol compared to treated gilts. No differences were observed between treatments in the 2nd clinical study. This lack of significant differences may be explained by the physiology of the porcine cycle and the sampling schedule. During the oestrus cycle, high circulating concentrations of estradiol bring gilts to oestrus [14], however, estradiol concentrations are only increased during a very short interval of approximately three to four days before ovulation [22] and, in the three studies summarized in this article, samples were taken every two to four weeks. Consequently, at these time points, we may miss the exact moment when estradiol release peaked, which would explain that significant differences in estradiol levels were observed only in the preclinical and the 1st clinical study.

According to the above results, administration of an anti-GnRF analogue is proven to induce antibodies against GnRF and results in temporary immunological suppression of ovarian function. This reduces the risk of unwanted pregnancy in gilts that may come in contact with sexually mature male pigs. Administration of an anti-GnRF analogue may also reduce aggressive and mounting behaviours of gilts when they are kept together [3, 23], resulting in lower levels of stress in individual animals, which makes them easier to handle in the farms and during transportation, and results in carcasses of better quality (less injuries, less lesions). These results have been proven in entire male pigs, but further research is needed to evaluate this in gilts [24]. Furthermore, in any open-air situation (like with Iberian females) where there is the need to prevent the sexual maturation of females with the intention to reduce attracting male wild boars (and therefore the potential transmission of infectious agents of major importance – such as African Swine Fever- from wild to domestic swine), the use of the anti-GnRF analog in females may be an additional benefit to replace the physical castration.

## Conclusions

Vaccination of gilts with anti-GnRF analog at 10 or 14 weeks of age, with a 2nd dose at 18 weeks (two different protocols) was effective in significantly reducing the onset of puberty for at least 9 weeks post 2nd vaccination. These results offer veterinarians and producers flexibility when implementing their vaccination programs adapted to pig management practices in commercial farms.

## Data Availability

No datasets were generated or analysed during the current study.
